# Post-treatment With Irisin Attenuates Acute Kidney Injury in Sepsis Mice Through Anti-Ferroptosis *via* the SIRT1/Nrf2 Pathway

**DOI:** 10.3389/fphar.2022.857067

**Published:** 2022-03-17

**Authors:** Zhang Qiongyue, Yang Xin, Peng Meng, Mi Sulin, Wang Yanlin, Li Xinyi, Song Xuemin

**Affiliations:** ^1^ Department of Anesthesiology, Zhongnan Hospital of Wuhan University, Wuhan, China; ^2^ Department of Cardiovascular Ultrasound, Zhongnan Hospital of Wuhan University, Wuhan, China; ^3^ Research Centre of Anesthesiology and Critical Care Medicine, Department of Anesthesiology, Zhongnan Hospital of Wuhan University, Wuhan, China

**Keywords:** ferroptosis, sepsis, irisin, acute kidney injury, SIRT1, Nrf2

## Abstract

Kidney is one of the most vulnerable organs in sepsis, resulting in sepsis-associated acute kidney injury (SA-AKI), which brings about not only morbidity but also mortality of sepsis. Ferroptosis is a new kind of death type of cells elicited by iron-dependent lipid peroxidation, which participates in pathogenesis of sepsis. The aim of this study was to verify the occurrence of ferroptosis in the SA-AKI pathogenesis and demonstrate that post-treatment with irisin could restrain ferroptosis and alleviate SA-AKI *via* activating the SIRT1/Nrf2 signaling pathway. We established a SA-AKI model by cecal ligation and puncture (CLP) operation and an *in vitro* model in LPS-induced HK2 cells, respectively. Our result exhibited that irisin inhibited the level of ferroptosis and ameliorated kidney injury in CLP mice, as evidenced by reducing the ROS production, iron content, and MDA level and increasing the GSH level, as well as the alteration of ferroptosis-related protein (GPX4 and ACSL4) expressions in renal, which was consistent with the ferroptosis inhibitor ferrostatin-1 (Fer-1). Additionally, we consistently observed that irisin inhibited ROS accumulation, iron production, and ameliorated mitochondrial dysfunction in LPS-stimulated HK-2 cells. Furthermore, our result also revealed that irisin could activate SIRT1/Nrf2 signaling pathways both *in vivo* and vitro. However, the beneficial effects of irisin were weakened by EX527 (an inhibitor of SIRT1) *in vivo* and by SIRT1 siRNA *in vitro*. In conclusion, irisin could protect against SA-AKI through ferroptotic resistance *via* activating the SIRT1/Nrf2 signaling pathway.

## Introduction

Sepsis is a systemic inflammatory reaction syndrome elicited by the host’s harmful response to infection. The morbidity of SA-AKI is closely correlated with the patient’s mortality in critical care units (Fani et al., 2018; Peerapornratana et al., 2019; Poston and Koyner, 2019). Sepsis is defined as a life-threatening organ dysfunction usually associated with severe renal dysfunction. As reported, there are 15% of septic patients who suffered from acute kidney injury (AKI) in America, while 38.4% of prevalence in Korea and Japan (Martin et al., 2003; Han et al., 2013; Shinjo et al., 2014). The pathogenesis of AKI in sepsis is extremely complex, and multi-factors work together, including vasoconstriction, reactive oxygen species formation, cell death, and regulation of abnormal inflammatory factors (Mehta et al., 2011; Basile et al., 2012). Therefore, SA-AKI constitutes a serious health problem which needs more attention.

Mounting evidence demonstrates that iron metabolism disorders play an important role in the AKI pathophysiologic process (Haase et al., 2010; Martines et al., 2013; Ige et al., 2019; Scindia PhD et al., 2019; Borawski and Malyszko, 2020; van Swelm et al., 2020). Ferroptosis is an iron-dependent programmed cell death (PCD), which is distinct from other types of cell death such as apoptosis, necrosis, pyrosis, and autophagy (Dixon et al., 2012). Its mechanism mainly involves intracellular iron overload and accumulation of reactive oxygen species (ROS) which lead to lipid peroxidation (Lei et al., 2019). Different from the traditional kind of PCD, the morphological traits of ferroptotic cells are characterized by smaller mitochondria, higher mitochondrial membrane density, disappearance of mitochondrial cristae, and rupture of the mitochondrial outer membrane (Yang and Stockwell, 2008; Dixon et al., 2012). The accumulation of lipid peroxides, especially phosphatidylethanolamine-ooh (PE-OOH), eventually leads to ferroptosis, and iron also seems to be a catalyzator or a principal regulator of ferroptosis (Kagan et al., 2017). Biochemically, the mechanism underlying ferroptosis is mainly related to glutathione (GSH) depletion, as well as the reduction of glutathione peroxidase 4 (GPX4) (Stoyanovsky et al., 2019). An amount of studies indicated that ferroptosis was involved in the occurrence and development of various diseases, including tumorigenesis, ischemia-reperfusion injury (IRI), brain and nerve diseases, kidney diseases, and hematonosis (Friedmann Angeli et al., 2014; Linkermann et al., 2014; Yang et al., 2014). Emerging researchers have confirmed system xc^–^ as a cysteine/glutamate antiporter is crucial in mediating the degree of ferroptosis, and the inhibition of system xc^–^ (such as erastin) can aggravate ferroptotic injury (Dixon et al., 2012; Lachaier et al., 2014; Kagan et al., 2017; Toyokuni et al., 2017). However, few open literature studies have reported the correlation between ferroptosis and SA-AKI.

As one of nicotinamide adenine dinucleotide-dependent histone deacetylase family members, silent information regulator transcript-1 (SIRT1) is a pivotal regulator in various processes, such as the processes of cell metabolism, senescent, apoptosis, inflammatory reaction, and oxidative stress (Kauppinen et al., 2013). The Nrf2 signal channel has been confirmed to mediate the activity and expression of system xc^–^ (Furfaro et al., 2016; Stockwell et al., 2017). Yang D et al. and Yang B et al. have proved that the SIRT1/Nrf2 pathway is related to antioxidative stress (Yang et al., 2016; Yang et al., 2018). It is confirmed that Nrf2 is an important negative regulator of ferroptosis in AKI. Up to this point, activating Nrf2 to inhibit ferroptosis seems to be a prospective target for the treatment of SA-AKI, which attracts us to find some therapies to protect against SA-AKI through activate Nrf2 to inhibit ferroptosis.

Irisin is a distinct hormone-like substance primarily secreted by the skeletal muscles during exercising (Benedini et al., 2017). Irisin promotes the white adipose tissues to become brown adipose tissue and helps to mediate lipid and glucose homeostasis (Yano et al., 2020). Previous studies have proved that irisin exerts antioxidative and anti-inflammatory actions in metabolic disorders (So and Leung, 2016; Özkur et al., 2020). Further studies indicated that irisin reduced ROS generation, preserved mitochondrial function, and exerted organ protection in the I/R-triggered heart, kidney, and liver injury (Wang et al., 2018; Bi et al., 2019; Zhang et al., 2021). Our previous research also proved that irisin could alleviate acute lung injury (ALI) (Li et al., 2019). Furthermore, whether irisin protects against SA-AKI through inhibition of ferroptosis or not remains unclear.

Therefore, we hypothesize that irisin could alleviate SA-AKI through upregulating the SIRT1/Nrf2 pathway. In this research, we have established the model of SA-AKI *in vivo* and *in vitro* to investigate the effects of irisin and shed light on the molecular mechanism that irisin retrained ferroptosis *via* activating the SIRT1/Nrf2 pathway.

## Methods and Materials

### Animals and Cells

All animals were purchased from the Animal Experimental Center of Wuhan University (ABLS-III Laboratory). C57BL/6 male mice weighing 20–25 g were used for this study. All mice were fed standard food and water and kept in a temperature- and humidity-controlled environment with a 12-h light–dark cycle. All mice were conducted following guidelines published by the NIH and approved by the Animal Ethics Committee of the Zhongnan Hospital of Wuhan University.

The HK-2 cells were obtained from the ATCC CRL-2190 standard (Bluefbio Co., Shanghai, China) and maintained in DMEM/F12 (Dulbecco’s modified Eagle’s medium; Gibco, United States) supplemented with 10% fetal bovine serum (FBS, DMEM, Gibco, Grand Island, United States) and 1% penicillin–streptomycin in an atmosphere with 5% CO_2_ at 37°C. All experiments were conducted following guidelines published by the NIH and approved by Wuhan University’s Ethics Committee.

### Experimental Model

Murine model of SA-AKI. The model of SA-AKI *in vivo* was set up, as previously recorded (Miyaji et al., 2003). All mice were anesthetized with administered pentobarbital (50 mg/kg, Sigma-Aldrich, MO, United States) intraperitoneally. Briefly, cecal ligation and puncture (CLP)-induced sepsis was established with a 22-gauge needle accordingly. The sham/Con group mice experienced the same operation without ligating or puncturing the cecum. Subsequently, pre-warmed normal saline with 1 ml per 25 g body weight was given subcutaneously. At 5 h after CLP, normal saline (NS, 0.5 ml, vehicle) or irisin (250 ug/kg, Phoenix Pharmaceuticals, Inc., Burlingame, 067-29A) was administered intravenously. Fer-1 (ferrostatin-1, 1.5 mg/kg, a ferroptosis inhibitor; Sigma-Aldrich, MO, United States), Fe (Fe-citrate, 15 mg/kg, a ferroptosis inducer; Sigma-Aldrich, MO, United States), or EX527 (0.1 ml, 10 mg/kg; a selective inhibitor of SIRT1; Santa Cruz Biotechnology) was administrated intravenously after irisin or NS injection, respectively. The doses of irisin, Fer-1, Fe, and EX527 were administered, as described previously (Wei et al., 2019; Qiang et al., 2020; Zhang et al., 2021). At 24 h after intravenous injection, animals were sacrificed *via* cervical dislocation. Following that, blood and kidney tissues were obtained for further experiments.

Cell model of SA-AKI. HK-2 cells were seeded into 96-well plates (5 × 10^5^ cells/well) and cultured for 24 h until 80% confluence. Subsequently, we have added LPS (10 μg/ml) into the cultured cells for 22 h to establish the cell model of LPS-induced AKI (Sun et al., 2021). First, to explore the reaction of ferroptosis, HK-2 cells were incubated with Fer-1 (0.1 μM) or Fe (3.3 mM) for 2 h before exposing to LPS for 22 h. The doses of Fer-1 and Fe were determined, as described previously (Qiang et al., 2020). Second, for detecting the role of irisin, the HK-2 cells were incubated with irisin (8 nM) before LPS administration. The dose of irisin was based on our pre-experimental study. Third, to confirm the function of irisin on ferroptosis, HK-2 cells were coincubated with irisin and Fe before LPS administration. Finally, to evaluate the role of SIRT1, negative control siRNA (si-con) or SIRT1 siRNA (si-Sirt1; a selective inhibitor of Sirt1 RNA, GenePharma Co., Ltd., Shanghai, China) was added into the HK-2 cells applying the lipofectamine 2000 TM reagent (Invitrogen, United States) for 48 h before LPS administration.

### Treatment Protocols

First, to detect the role of irisin in ferroptosis in SA-AKI, 60 WT mice were randomly allocated into six groups (*n* = 10): the sham group, CLP group, Fer-1 (CLP + Fer-1) group, Fe (CLP + Fe) group, Ir (CLP + Irisin) group, and Ir + Fe (CLP + Irisin + Fe) group. Second, to demonstrate the protective reaction of irisin on SA-AKI by regulating the SIRT1/Nrf2 channel, 30 WT mice were randomly allocated into five groups (*n* = 6), respectively: the sham group, CLP group, Ir (CLP + Irisin) group, EX527 (CLP + EX527) group, and Ir + EX527 (CLP + Irisin + EX527) group.

### Evaluation of Serum Samples

To evaluate renal function, serum BUN and Cre concentrations were detected by the colorimetric and the creatinine assay kit (Nanjing Jiancheng, China), according to standard methods, respectively. NGAL concentration was measured by using the human lipocalin-2/NGAL ELISA kit (Boster Biological Technology Ltd., Wuhan, China) according to standard methods.

### Histopathological Analysis

The left renal samples were dissected and fixed with paraformaldehyde. Next, renal samples were paraffin-embedded, sliced into 4-μm slices, and stained with hematoxylin and eosin (H&E) (Chen et al., 2011). Finally, the acute tubular necrosis score (ATN score) was quantitated by calculation of the percent of tubules from 10 randomly chosen, none overlapping fields (200×) that displayed cell necrosis, loss of brush border, cast formation, and tubule dilatation as follows: 0, none; 1, ≤10%; 2, 11–25%; 3, 26–45%; 4, 46–75%; and 5, >76% (Chen et al., 2011). Evaluation of the specimens was performed in a blinded manner to the mice group.

### Transmission Electron Microscopy

The left kidney samples were excised and fixed in glutaraldehyde. Then, the fixed tissues were dehydrated with a grade ethanol series and sliced into ultrathin slices. Afterward, the slices were stained with uranyl acetate and lead citrate. Finally, the slices were detected with an HT-7500 transmission electron microscope (Hitachi. Co., Japan) (Li et al., 2021).

### Detection of ROS Levels

The levels of ROS from renal tissues were assessed using the dihydroethidium (DHE) fluorescent probe (D7008, Sigma-Aldrich, MO, United States), following the previously described protocols (Fan et al., 2021). In brief, after frozen sections were incubated with 50 uM DHE for 1 h in the dark at room temperature, the sections were incubated for 10 min with DAPI (1 mg/ml). The cell images were taken with a fluorescent microscope (TE-2000, Nikon, Co., Japan) with an excitation (Ex) wavelength at 525 nm and an emission (Em) wavelength at 610 nm after washing. The values used ImageJ software to capture the red (the oxidized probe) and blue (the reduced probe) fluorescence intensities to calculate the ratio and then standardized to the con group.

The levels of ROS in HK-2 cells were tested by the 2′, 7′- dihydrofluorescein diacetate (DCFH-DA) fluorescent probe (D6883, Sigma-Aldrich, United States), following the manufacturer’s protocols. Briefly, after treating HK-2 cells with 10 uM DCFH-DA in the dark for 1 h, the pictures were detected using a fluorescent microscope (TE-2000, Nikon, Co., Tokyo, Japan) at (Ex/Em) 485 nm/530 nm.

### Enzyme-Linked Immunosorbent Assay

The levels of TNF-α, IL-1β, and IL-6 in kidney tissues were detected by using the TNF-α ELISA kit (CSB-E04741m, Wuhan, China), IL-1β ELISA kit (CSB-E16568G, Wuhan, China), and IL-6 ELISA kit (CSB-E04639m, Wuhan, China), according to the instructions of respective manufacturer.

### Determination of Related Indicators

The concentrations of malondialdehyde (MDA), glutathione (GSH), and ferrous ions in tissue lysates and HK-2 cells were determined by the MDA assay kit (Beyotime, Wuhan, China), GSH assay kit (Beyotime, Wuhan, China), and iron assay kit (MAK025, Sigma-Aldrich, MO, United States), following the respective manufacturer’s protocols.

### Western Blot

We performed Western blot, as previously recorded ((Zhang et al., 2016; Fei et al., 2020)). The proteins extracted from renal tissues or cells were lysed in a RIPA buffer containing protease and phosphatase inhibitors, and the protein content was detected by using the BCA protein assay kit (Beyotime). Protein samples (50 ug) from each group were electrophoresed in 10% SDS-PAGE gel and then transferred to a PVDF membrane. After blocking with 5% nonfat dry milk, the blots were incubated with the primary antibodies against GPX4 (1: 1000, DF6701, Affinity), ACSL4 (1: 1000, A14439, Ablonal), Nrf2 (1: 1000, AF7904, Affinity), SIRT-1 (1: 1000, DF6033,Affinity), and or β-actin (1: 200, ab181602, Abcam) overnight at 4°C. Subsequently, the blots were washed in TBST and incubated with a secondary antibody for 2 h at room temperature. The bands were determined with an ECL system applying an ECL kit (Applygen), and band intensities were checked by BandScan software.

### Detection for Cell Viability

The CCK-8 assay kit (Beyotime) was used to detect the cell viability, as previously illustrated (Li et al., 2018). Briefly, before being subjected to various treatments, the HK-2 cells were added to a density at 6 × 10^3^ cells per well 96-well plates and incubated for 24 h. Following that, each well was added 10 ul of the CCK8 solution and cultivated for an additional 3 h. The absorbance was measured by using a microplate reader (PerkinElmer, United States) at 450 nm.

### Assay for Lipid ROS

Lipid ROS was detected using the BODIPY^TM^ 581/591 C11 (D3861, Invitrogen) fluorescent probe, as previously illustrated (Yagoda et al., 2007; Song et al., 2018; Wang et al., 2021). First, HK-2 cells were treated for 1 h at 37°C with 2 uM of BODIPY 581/591 C11. Second, the cells were then rinsed in PBS, digested with trypsin, and suspended in PBS. At last, cell images were taken by flow cytometry (BD Biosciences, BD Accuri C6 plus, United States).

### Mitochondrial Membrane Potential (MMP) Assay

The MMP of HK-2 cells was measured by using the JC-1 fluorescent probe (CAS 3520-43-2, Abcam), according to the instructions of the manufacturer (Burbridge et al., 2021). Cells were treated with 5 μM JC-1 in the dark for 30 min at 37°C. After washing with PBS, cell images were taken by using a fluorescent microscope (TE-2000, Nikon, Co., Japan).

### Mitochondrial Ferrous Iron (Fe^2+^) Fluorescence Probe

The Mito Tracker Green fluorescent probe (Beyotime, China) was applied to examine the mitochondrial Fe^2+^ of HK-2 cells, according to the instructions of the manufacturer (Qi et al., 2012). Concise and to the point, the medium was discarded, and the cells were treated with 5 μM Mito FerroGreen working liquid for 30 min in the dark. After that, the working fluid was removed, and then, the fresh DMEM was added. Finally, a confocal microscope (TCS-SP2, Leica, Germany) was used to examine the cell images at Ex and Em wavelengths of 488 nm and 510–550 nm, respectively.

### Statistical Analysis

All data are shown as means ± standard deviation. Data are expressed as means ± standard deviation (SD) of the indicated number of independent experiments. Differences among multiple groups were analyzed by one-way ANOVA, followed by Tukey’s post-tests. Statistical analysis was determined by SPSS software 17.0 (SPSS, Chicago, IL) and then drawn with GraphPad Prism software (ver. 8.0.1; GraphPad, San Diego, CA, United States). *p* < 0.05 was considered as a statistical significance.

## Results

### Irisin Treatment Protects Against Acute Kidney Injury in the CLP-Induced Sepsis Model

**FIGURE 1:**
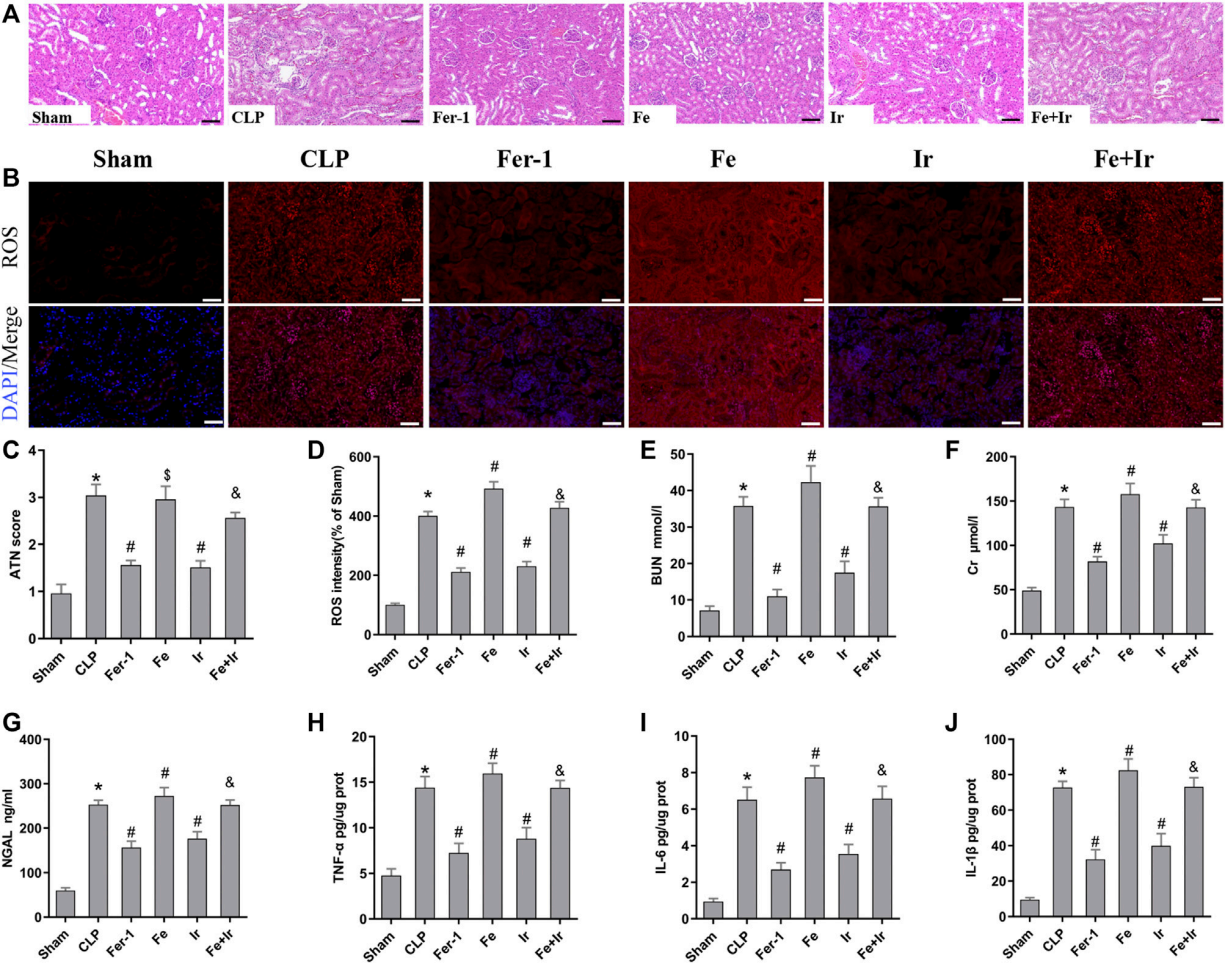
Irisin attenuates CLP-induced kidney injury. Mice were given a sham operation or CLP model operation, with or without irisin (Ir), ferrostatin-1 (Fer-1), or/and Fe-citrate (Fe) (Fe + Ir) administration. **(A)** Representative images (200×) showing HE staining of renal sections (scale bar = 50 μm). **(B)** Representative fluorescent images of DHE staining (scale bar = 50 μm). **(C)** Acute tubular necrosis score (ATN score). **(D)** The quantification of the DHE fluorescence intensity. **(E)** BUN concentration in the plasma. **(F)** Creatinine (Cr) concentration in the plasma. **(G)** Plasma neutrophil gelatinase-associated lipocalin (NGAL) concentration. **(H–J)** The level of TNF-α, IL-6, and IL-1β in the kidney tissue. *p < 0.05 vs. Sham group, *p* < 0.05 vs. CLP group, P <0.05 vs. Fe group, and *p* > 0.05 vs CLP group. Data are presented as mean ± SD (*n* = 10).

To confirm the role of ferroptosis in SA-AKI, we explored the impact of Fer-1 administration. As shown in Figures 1A,C, SA-AKI produced significant pathological changes, including proximal tubule dilation, brush border damage, proteinaceous casts, interstitial widening, and necrosis. ATN can reflect the degree of renal pathological injury (Chen et al., 2011). However, Fer-1 treatment alleviated sepsis-induced pathological changes (Figures 1A,C). The serum BUN, SCr, and NGAL levels were determined to represent the renal function, as previously recorded (Wei et al., 2006). As illustrated in Figures 1E–G, administration of Fer-1 significantly mitigated sepsis-stimulated kidney injury, as evidenced by BUN, Cr, and NGAL. To confirm the injury indeed happened in the kidney while sepsis happened, we detected TNF-α, IL-6, and IL-1β in the kidney tissue, as shown in Figures 1H–J. These results confirmed that ferroptosis occurred in SA-AKI.

Resembling the effects of Fer-1 administration, irisin post-conditioning also lessened SA-AKI. (Figures 1A,C, Figures 1E–G). However, iron supplementation with Fe-citrate exacerbated SA-AKI, resulting in severe pathological lesions and impairment of function, which were attenuated by irisin (Figures 1A,C, Figures 1H–J).

### Irisin Attenuates Ferroptosis During SA-AKI

**FIGURE 2:**
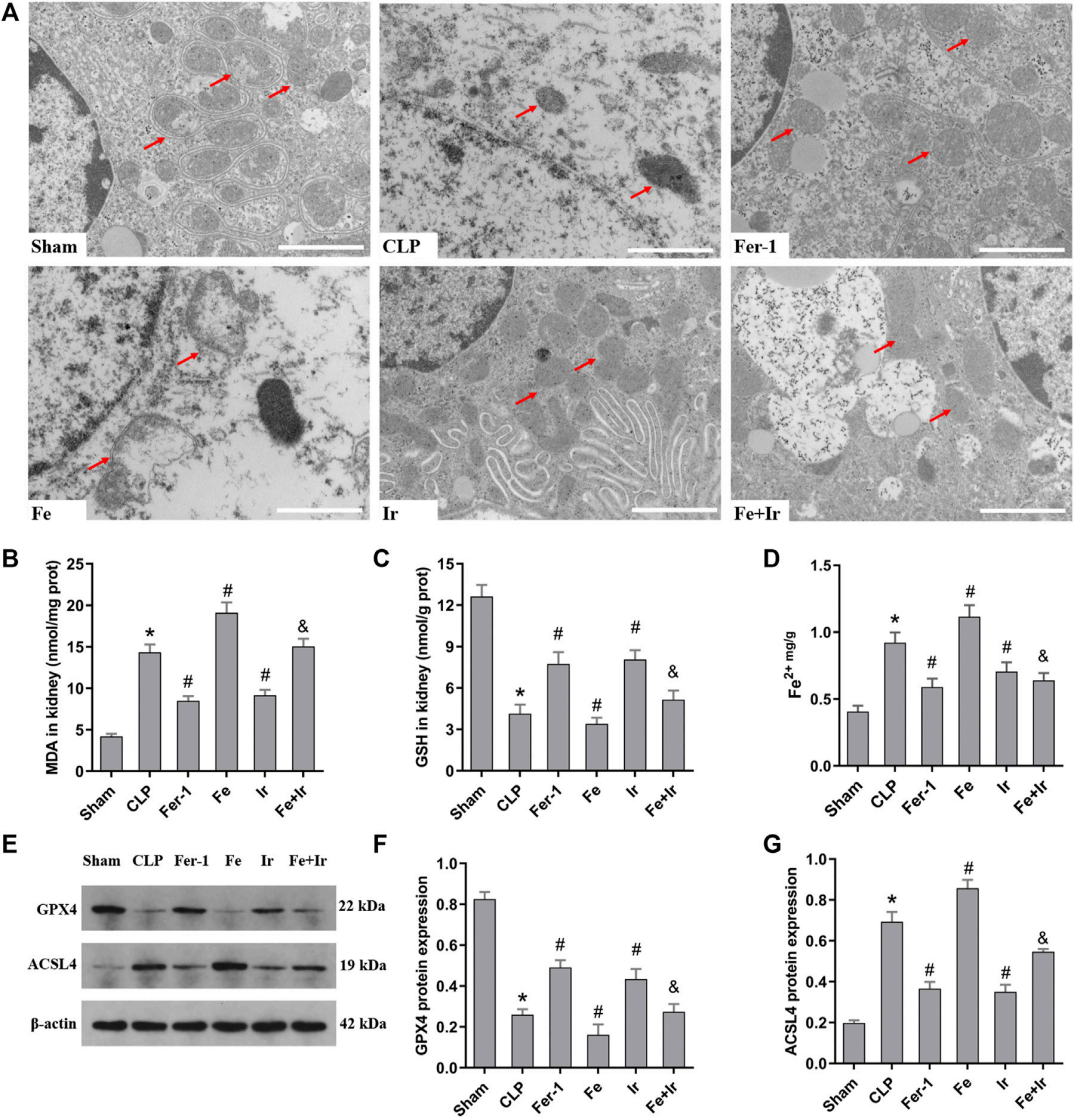
Irisin alleviates the occurrence of ferroptosis in SA-AKI. **(A)** Representative TEM images (scale bar = 1 μm, upper panels). The red arrow indicates representative mitochondria in mouse kidney (n = 3 mice/group). The levels of MDA **(B)**, GSH **(C)**, and Fe2+ **(D)** in mouse kidney homogenates. (*n* = 10). **(E)** Western blot analysis of GPX4 and ACSL4 proteins in the kidney tissue. (*n* = 3). **(F,G)** Quantitation of results in **(E)**. *p < 0.05 vs. Sham group, ^#^ *p* < 0.05 vs. CLP group, ^&^ *p* < 0.05 vs. Fe group. Data are presented as mean ± SD.

To further prove that the protection of irisin on SA-AKI referred to ferroptosis, the tissue ROS production and lipid ROS level (MDA and GSH) were measured. SA-AKI markedly increased the generation of ROS and MDA levels and decreased the GSH activity, whereas Fer-1 and irisin post-conditioning inhibited the oxidative damage caused by sepsis (Figures 1B,D, Figures 2B,C). Next, the iron content and key ferroptosis-related protein (GPX4 and ACSL4) expressions were determined. The data showed that the iron content and the expression of the pro-ferroptotic protein ACSL4 increased, while the anti-ferroptotic protein GPX4 expression reduced during sepsis. However, these alterations were suppressed by Fer-1 and irisin post-conditioning (Figures 2D–G). The morphological features of ferroptosis were assessed *via* TEM. Accordingly, sepsis induced significant morphological changes, including smaller mitochondria and cristae reduction, whereas Fer-1 and irisin alone ameliorated sepsis-induced mitochondrial morphologic changes (Figure 2A).

Overall, these data suggested that ferroptosis contributed to SA-AKI, and irisin post-conditioning mitigated SA-AKI through inhibiting ferroptosis.

### Irisin Mitigates Ferroptosis and Mitochondria Dysfunction in LPS-Induced HK2 Cells

**FIGURE 3:**
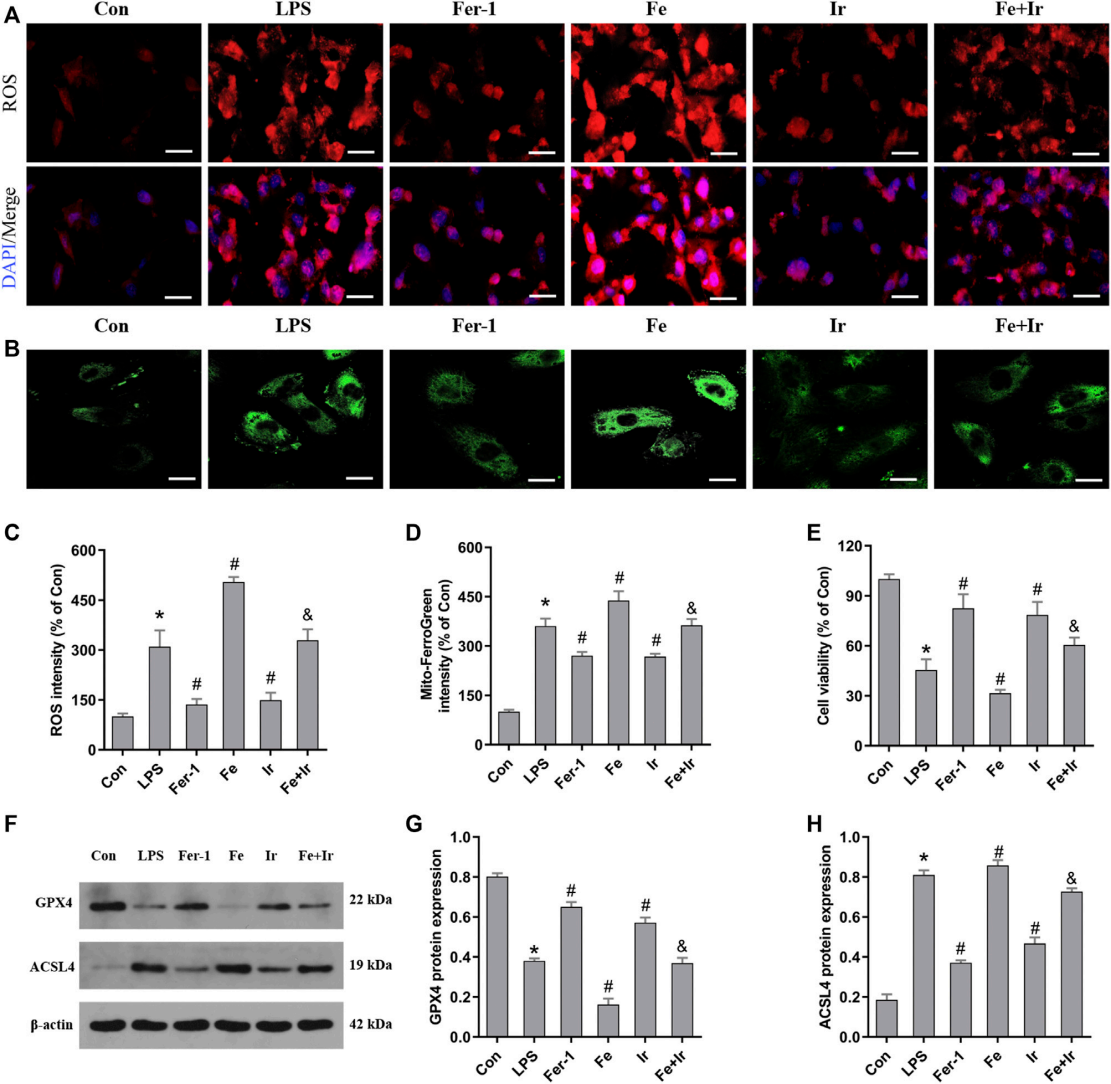
Irisin inhibits LPS-induced ferroptosis in HK-2 cells. **(A)** Images of intracellular ROS levels in HK-2 cells stained by DCFH-DA (10 μM) fluorescent probes (Bar = 50 μm). **(B)** Fluorescence images of Mito FerroGreen (5 μM)-stained HK-2 cells (Bar = 20 μm). **(C)** Quantitative results of ROS. **(D)** Quantitative results of mitochondrial ferrous iron. **(E)** Cell viability was determined by CCK-8 in HK-2 cells. **(F)** Western blot analysis of GPX4 and ACSL4 proteins in HK-2 cells. **(G,H)**, Quantitation of results in **(F)**. *p < 0.05 vs. Con group, ^#^ *p* < 0.05 vs. LPS group, ^&^ *p* < 0.05 vs. Fe group. Data are presented as mean ± SD (*n* = 3).

Based on the aforementioned findings *in vivo*, we next evaluated the function of irisin on LPS-induced AKI. As shown in Figure 3E, irisin and Fer-1 alone blocked the loss of cell death caused by LPS. The measurement of intracellular ROS using the DCFH-DA fluorescent probe showed that Fer-1 and irisin post-conditioning decreased ROS levels in HK-2 cells after LPS-induced sepsis, while Fe transfection increased ROS levels (Figures 3A,C). Similar to Fer-1 alone, irisin post-conditioning inhibited the LPS-induced increase of the ACSL4 expression and prevented the decrease of the GPX4 expression (Figures 3F–H). The results of Mito FerroGreen staining consistently indicated that Fer-1 and irisin have a beneficial effect on LPS-induced injury, while Fe exacerbated LPS-induced injury (Figures 3B,D). Notably, irisin abrogated the effect of Fe on LPS-induced injury (Figures 3A–H). Our research data have revealed that irisin could inhibit LPS-induced ferroptosis *in vitro*.

### Irisin Inhibits LPS-Induced Ferroptosis Through the SIRT1/Nrf2 Pathway *In Vitro*

**FIGURE 4:**
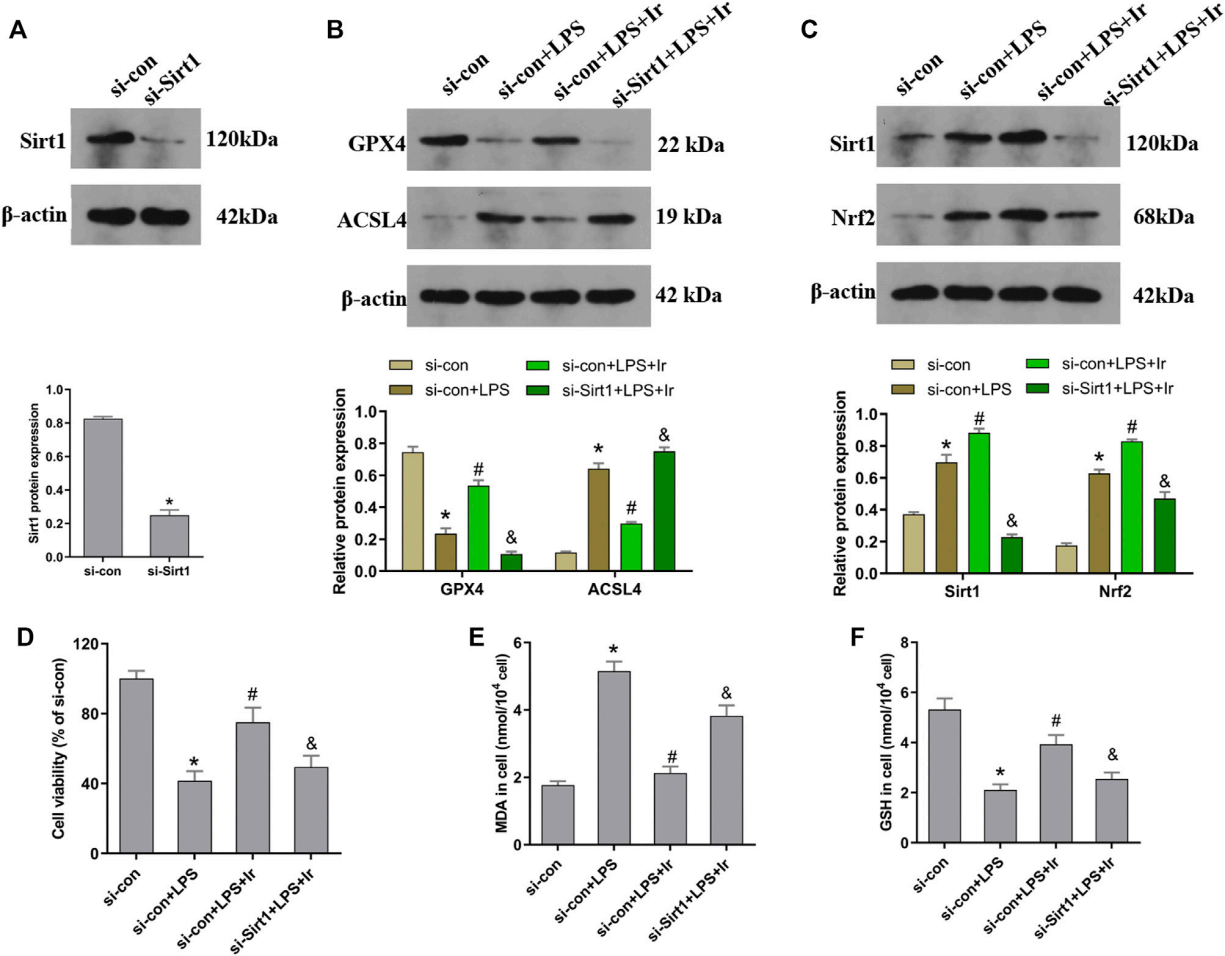
Irisin upregulates the SIRT1/Nrf-2 pathway to inhibit LPS-induced ferroptosis. HK-2 cells were transfected with SIRT1 siRNA (si-Sirt1) or negative control (si-con) followed by exposure to LPS and irisin. **(A)** The expression of SIRT1 protein in cells transfected with SIRT1 siRNA (si-Sirt1) or negative control (si-con) was detected using Western blot to validate the knockdown of SIRT1. **(B)** Western blots and quantitative analyses of GPX4 and ACSL4 proteins in cells. **(C)** Western blots and quantitative analyses of SIRT1 and Nrf2 proteins in cells. **(D)** Cell viability using the CCK-8 kit. The levels of MDA **(E)** and GSH **(F)** in cells were analyzed using a corresponding kit. *p < 0.05 vs. si-con group, ^#^ *p* < 0.05 vs. si-con + LPS group, ^&^ *p* < 0.05 vs. si-con + LPS + Ir group. Data are presented as mean ± SD (*n* = 3).

To explore the function of the SIRT1/Nrf2 axis in irisin-mediated protection on LPS-induced injury, we used si-SIRT1 to knock down the SIRT1 expression. As exhibited in Figure 4A, the expression of SIRT1 was markedly decreased after transfection. The SIRT1 and Nrf2 protein expressions were analyzed using the Western blot technique. As displayed in Figure 4C, irisin markedly upregulated the level of SIRT1 and Nrf2 in HK2 cells. Irisin post-conditioning could suppress the decrease in the cell viability and oxidative damage caused by LPS, while si-SIRT1 transfection eliminated this effect of irisin (Figures 4D–F). Consistently, the si-Sirt1 transfection was able to reverse the effect of irisin on the alteration of ACSL4 and GPX4 expressions induced by LPS (Figure 4B).

**FIGURE 5:**
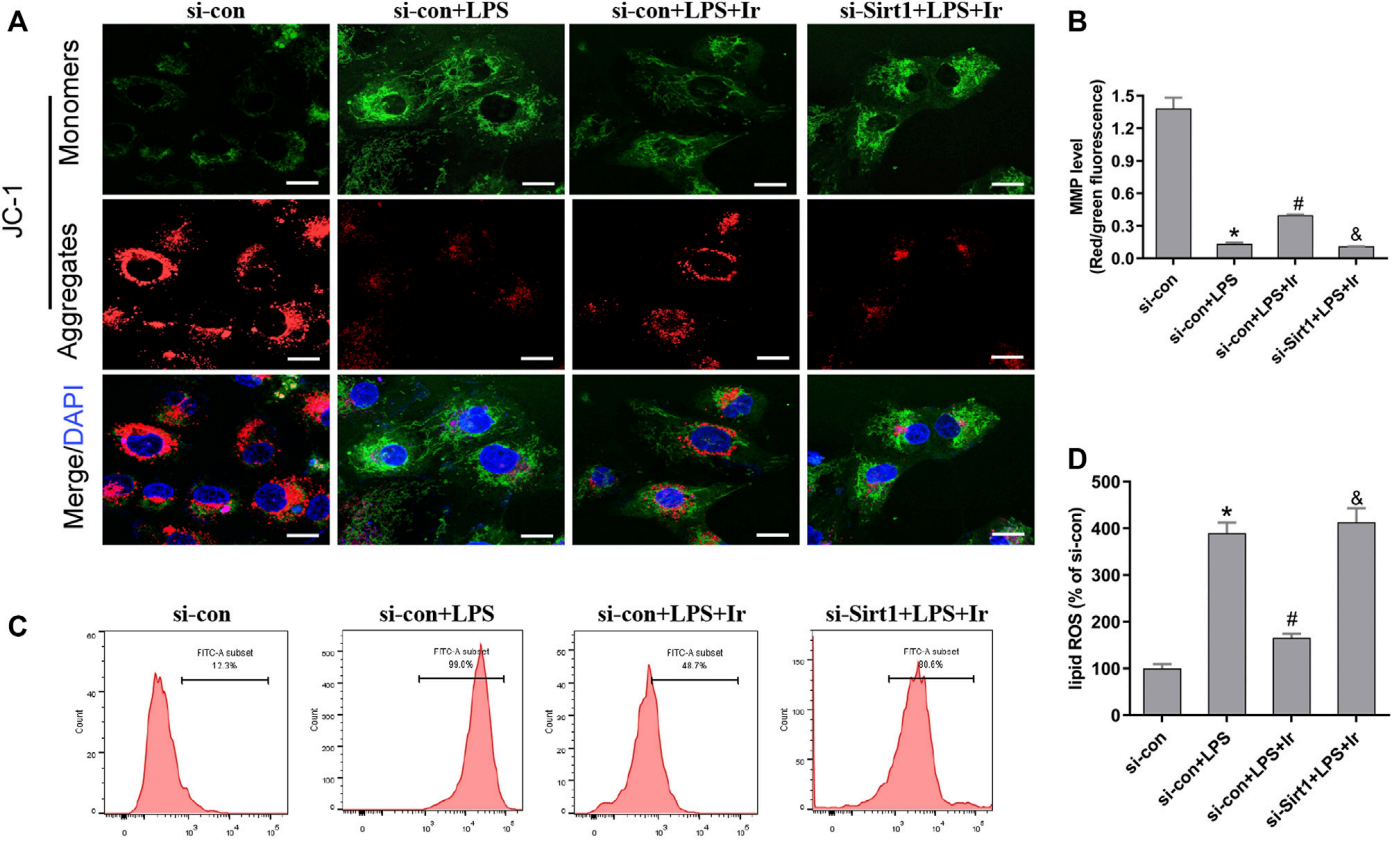
Irisin inhibits LPS-induced accumulation of ROS and lipid peroxidation. **(A)** Fluorescence images of 5 μM JC-1-stained HK-2 cells. Scale bars: 20 μm. The ratio of red and green fluorescence reflected changes of the mitochondrial membrane potential (MMP) (*n* = 3). **(B)** Quantitative results of MMP. **(C)** lipid ROS was analyzed using 2 μM BODIPY® 581/591 C11 by flow cytometry (n = 5). **(D)** Quantitative results of lipid ROS (n = 3). *p < 0.05 vs. si-con group, ^#^ *p* < 0.05 vs. si-con + LPS group, ^&^ *p* < 0.05 vs. si-con + LPS + Ir group. Data are presented as mean ± SD (*n* = 3).

Mitochondria are the central role of redox homeostasis and play a significant role in the pathological process of ferroptosis. MMP assays indicated that irisin has an advantageous impact on LPS-induced MMP reduction, while si-SIRT1 transfection reverses this effect (Figures 5A,B). The results of lipid ROS also indicated that the si-SIRT1 transfection reversed the protective effect of irisin on lessening lipid ROS accumulation stimulated by LPS (Figures 5C,D). The aforementioned results implied that irisin inhibited LPS-stimulated ferroptosis through activating the SIRT1/Nrf2 pathway.

### Irisin Attenuates SA-AKI Through the SIRT1/Nrf2 Pathway *In Vivo*

**FIGURE 6:**
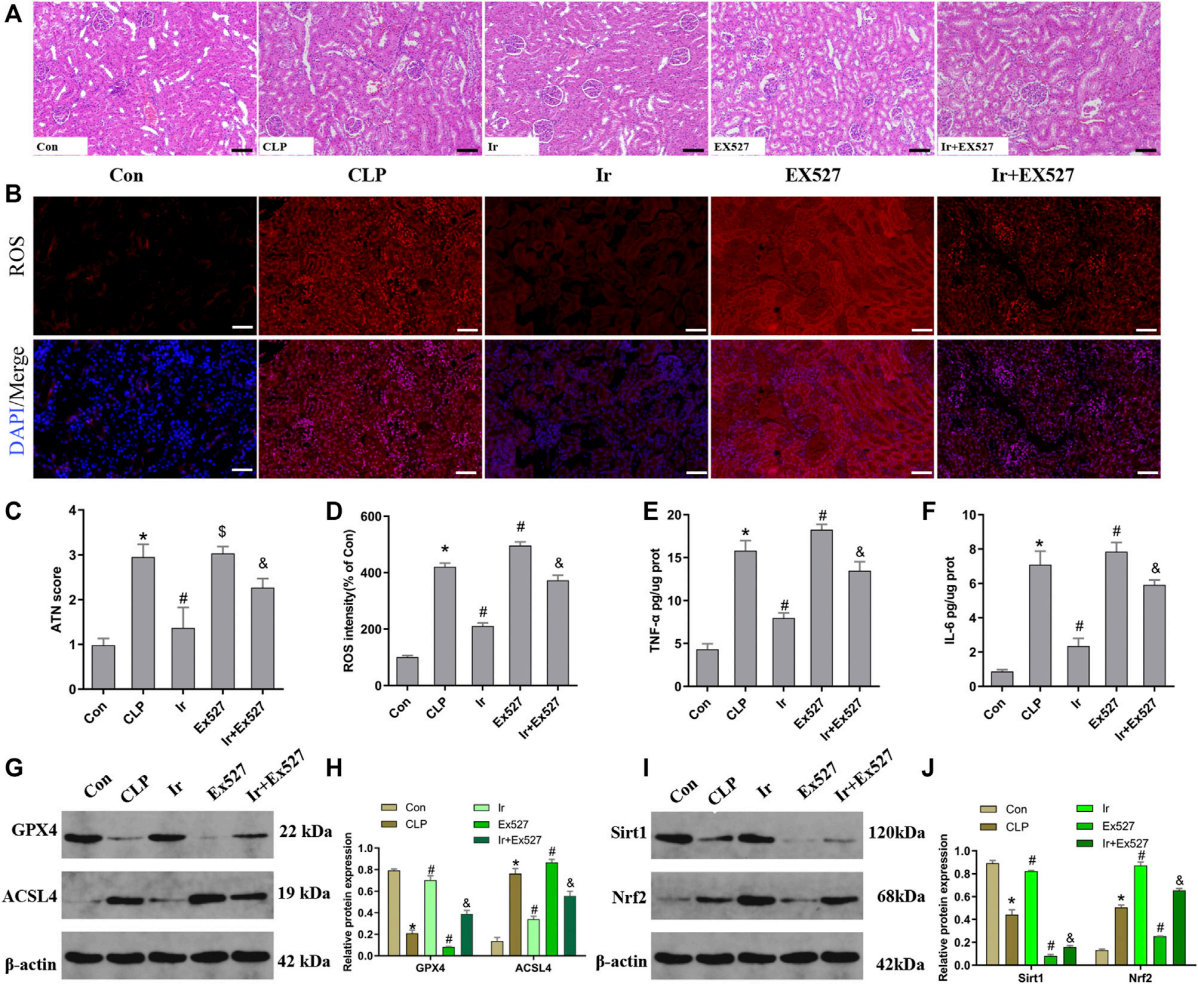
Irisin attenuates CLP-induced kidney injury through the SIRT1/Nrf-2 pathway. Mice were given a sham operation or CLP model operation, with or without irisin (Ir) and Ex527 administration. **(A)** Representative images (200×) showing HE staining of renal sections (scale bar = 50 μm). **(B)** Representative fluorescent images of DHE Staining (scale bar = 50 μm). **(C)** Acute tubular necrosis score (ATN score). **(D)** The quantification of DHE fluorescence intensity. The level of TNF-α **(E)** and IL-6 **(F)** in the kidney tissue. **(G)** Western blot analysis of GPX4 and ACSL4 proteins in the kidney tissue. (*n* = 6). **(H)** Quantitation of results in **(G)**. **(I)** Western blot analysis of SIRT1 and Nrf2 proteins in the kidney tissue. **(J)** Quantitation of results in **(I)**. *p < 0.05 vs. Con group, ^#^ *p* < 0.05 vs. CLP group, ^&^ *p* < 0.05 vs. CLP + Ir group, ^$^ *p* > 0.05 vs CLP group. Data are presented as mean ± SD (*n* = 6).

**FIGURE 7:**
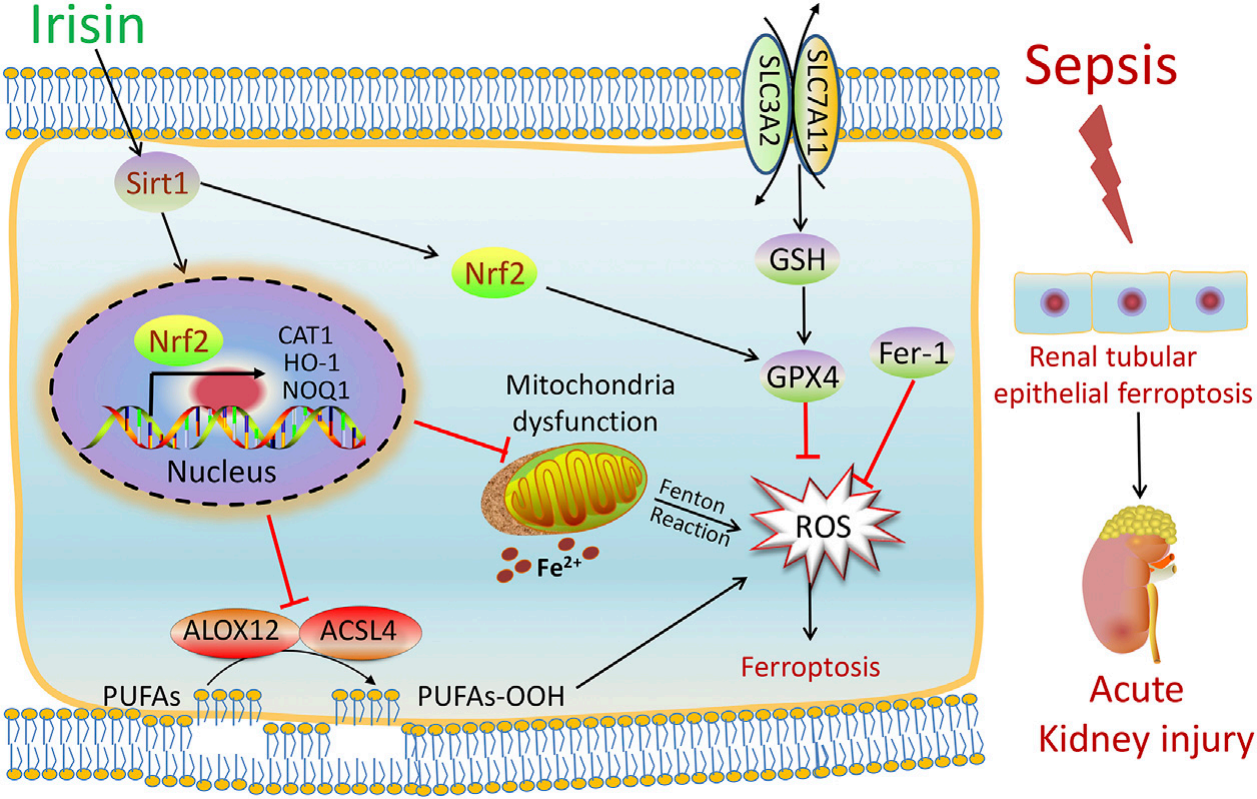
Scheme summarizing the protective effects of irisin post-treatment on sepsis-associated acute kidney injury (SA-AKI) *via* induction of the SIRT1/Nrf2 signal axis. SA-AKI leads to increased iron and lipid peroxidation, along with GSH depletion, which is implicated in the pathological process of SA-AKI. Irisin post-treatment effectively suppressed ferroptosis and alleviated SA-AKI and improved mitochondrial function *via* induction of the SIRT1/Nrf2 signal axis. The ferroptosis-specific inhibitor ferrostatin-1 (Fer-1) and the ferroptosis inducer Fe-citrate (Fe) could attenuate or exacerbate ferroptosis in SA-AKI, respectively.

To further investigate the function of the SIRT1/Nrf2 axis in irisin-mediated protection on SA-AKI *in vivo*, we used the EX527 to inhibit SIRT1 *in vivo*. As shown in Figures 6A–J, irisin post-conditioning could attenuate the kidney damage caused by CLP, while EX527 treatment eliminated this beneficial effect of irisin (Figures 7A–J). The measurement of intracellular ROS using the DCFH-DA fluorescent probe showed that irisin decreased ROS levels in mice after sepsis. The results of renal ROS also indicated that the silence of the SIRT1/Nrf2 axis reversed the protective impact of irisin on lessening lipid ROS accumulation stimulated by sepsis (Figures 6B,D). Consequently, these results further supported that the protection of irisin on SA-AKI stimulated ferroptosis through the SIRT1/Nrf2 pathway.

## Discussion

The current research focused on the role of irisin in ferroptosis and its possible therapeutic effect on SA-AKI. The major findings of this study are as follows (Peerapornratana et al., 2019): ferroptosis is involved in SA-AKI; (Poston and Koyner, 2019); irisin post-treatment protects against SA-AKI by inhibiting ferroptosis; (Fani et al., 2018); the protective mechanism of irisin on SA-AKI depends on the SIRT1/Nrf2 pathway.

The kidney is one of the most vulnerable organ responses to a sepsis-induced inflammatory reaction. The occurrence of renal injury increases the average hospital stay and mortality of septic patients (Peerapornratana et al., 2019; Poston and Koyner, 2019). Many studies indicate that acute kidney injury is followed by the generation of ROS and the occurrence of lipid peroxidation (3, 10). Ferroptosis is discovered as a novel cell death type, which is an iron-dependent, lipid peroxidation-induced cell death cascade (Dixon et al., 2012). Accumulating studies found that the catalyst regulator and the critical initiator of ferroptosis is iron, and enhanced iron uptake, decreased iron reserve, dysfunction of iron exporters, or disruption of ferritin may result in iron overload (Dixon et al., 2012; Lei et al., 2019). In recent years, researchers have found complex cellular networks that induce oxygen-free radical production and suppress lipid peroxidation (Lei et al., 2019). The mechanism of ferroptosis has been found mediated through two major pathways, such as decreased reduction of lipid peroxides caused by the inhibition of GPX4 and the increased production of lipid peroxides from arachidonic acid (AA) (Lei et al., 2019). Meanwhile, some key genes also take part in the development of ferroptosis, such as ACSL4, which inserts unsaturated AA into the cellular membrane, and GPX4, which eliminates lipid peroxides by utilizing GSH (Hadian and Stockwell, 2020). Some biomarkers, including the iron content, levels of lipid peroxides, ferroptosis-related protein (i.e., ACSL4 and GPX4) expressions, and mitochondrial morphological characteristics, are often recognized to estimate ferroptosis. In our study, SA-AKI or LPS-induced AKI caused apparent iron accumulation, promoted ROS-induced lipid peroxidation, and resulted in severe mitochondrial lesions in mice and HK-2 cells. Consistently, the expression of ACSL4 was elevated, while the expression of GPX4 was reduced under the conditions of SA-AKI or LPS-induced AKI. These findings were in line with previous research studies on sepsis-related injuries (Martines et al., 2013; Borawski and Malyszko, 2020). Some studies showed that inhibiting ferroptosis by Fer-1 could alleviate the kidney injury induced by I/R, whereas ferroptosis inducer Fe treatment could exacerbate this injury (Scindia PhD et al., 2019; van Swelm et al., 2020). Consistent with this study, we also found that Fer-1 or Fe treatment could attenuate or aggravate SA-AKI, which further demonstrated that ferroptosis occurred during SA-AKI, and inhibition of ferroptosis may provide a promising strategy for the prevention of SA-AKI.

Irisin is a new member of exercise-induced metabolic hormone discovered in 2012, which contributes to inducing the browning of white adipocytes, improving energy metabolism, and glucose utilization (Boström et al., 2012). Therefore, irisin may become an attractive pharmacological target for metabolic diseases (So and Leung, 2016; Askari et al., 2018; Yano et al., 2020; Özkur et al., 2020). Recently, the antioxidative effect of irisin has attracted substantial attention. Mounting research practices have documented that the raised level of irisin in the plasma is correlated with reduced oxidative stress and the enhanced antioxidant system in various models of I/R such as the heart, liver, and kidney (Wang et al., 2018; Bi et al., 2019; Zhang et al., 2021). The concentration of irisin in the plasma has been demonstrated to be reduced in septic patients and animals with sepsis (Wei et al., 2020). However, there are no available data about the effect of irisin post-conditioning on SA-AKI. In this study, we confirmed that irisin effectively reduced the production of ROS and lipid peroxidation, while increasing the levels of antioxidant enzymes GSH following SA-AKI, which indicated that irisin could alleviate sepsis-associated kidney injury-induced oxidative damage. Notably, we observed that irisin efficiently decreased the intracellular iron content, increased the GPX4 expression, and reduced the ACSL4 expression following SA-AKI. Based on these investigations, we presumed that the antioxidant activity of irisin on SA-AKI might relate to the inhibition of ferroptosis. To prove this hypothesis, we have used Fe to take rescue experiments. As we expected, the protective effects of irisin were eliminated by Fe. These results indicated that the efficacy of irisin is mainly achieved by inhibiting ferroptosis.

As an important organelle in the cytoplasm, mitochondria play a significant role in regulating metabolisms, such as ATP production, intracellular calcium homeostasis, ROS production, and apoptosis. Mitochondrial dysfunction is firmly connected with sepsis-induced organ dysfunction (McCreath et al., 2016). Mitochondria are the core of redox homeostasis and participate in regulating iron homeostasis and cell metabolism during ferroptosis (Wang et al., 2020). Mitochondrial dysfunction and metabolic changes induced by diverse intracellular and extracellular stimuli determine the fate of cells. Various cellular metabolic pathways, including amino acid, iron, and lipid metabolism, can initiate ferroptosis (Wang et al., 2020). Mountain evidence suggests that the association between mitochondria and ferroptosis is adjusted by the alteration of the mitochondrial membrane potential (MMP) and the regulation of the mitochondrial permeability transition pore (MPTP) (Gao et al., 2019). In this study, we monitored the mitochondrial function *via* the MMP assay and fluorescence probe. Our results showed that SA-AKI led to a decrease in the MMP and increased mitochondrial Fe^2+^ in HK-2 cells. However, irisin reversed the decline in the MMP and reduced the mitochondrial iron level. These findings indicated that the mitochondrion was an essential player in the anti-ferroptosis of irisin.

SIRT1 is a nicotinamide adenine dinucleotide (NAD^+^)-dependent histone deacetylase, which is a member of the mammalian sirtuin family, closely associated to biological processes such as cell metabolism, aging, apoptosis, inflammation, and oxidative stress (Alcendor et al., 2007; Hsu et al., 2010; Kauppinen et al., 2013; Yang et al., 2016; Dodson et al., 2019). Numerous studies provided that SIRT1 is regarded as a critical regulator in inflammation, apoptosis, and antioxidant defense systems (Alcendor et al., 2007; Hsu et al., 2010; Planavila et al., 2011). In addition, a number of research practices have also demonstrated that SIRT1 could upregulate the Nrf2 expression to reduce the production of ROS (Huang et al., 2013; Yang et al., 2016; Prola et al., 2017; Yang et al., 2018). Emerging pieces of evidence prove that Nrf2 is a key regulator for the function of system xc^−^ while excessive oxidative stress depletes a large amount of Nrf2, disrupting homeostasis (Yang et al., 2016; Stockwell et al., 2017; Hadian and Stockwell, 2020). In this study, our protein expression data indicated that irisin upregulated the expression of SIRT1 and Nrf2 during SA-AKI. The advantageous effect of irisin on SA-AKI and mitochondria protection were reversed by si-SIRT1 and Ex527, which further indicated that irisin elicited kidney protection by activating the SIRT1 signaling pathway. Additionally, SIRT1 knockdown by using si-SIRT1 and Ex527 significantly inhibited irisin-induced SIRT1 expression and reduced the Nrf2 expression, suggesting that Nrf2 induction may be mediated by SIRT1 activation.

Despite these findings, our research has several limitations. First, SA-AKI can affect renal tubular epithelial cells, as well as glomerular cells, and renomedullary interstitial cells. However, our *in vitro* experiments were limited to renal tubular epithelial cells. Second, we focused on the effect of irisin on ferroptosis. Although we determined the direct anti-ferroptosis impacts of irisin by familiar modulators of ferroptosis such as Fer-1 and Fe-citrate, we did not detect the effect of irisin in other types of cell death such as autophagy and necroptosis. In addition, in our research, the expression of SIRT1 in the sham group *in vivo* and in the con group *in vitro* has significant differences; the reason maybe that there are many differences in regulatory mechanisms between *in vivo* and *in vitro* experiments. This difference may be due to the influence of other kidney cells, other organs, or hormone levels on the expression of SIRT1 *in vivo* experiments. However, the cells used in *in vitro* experiments are a single variety, and the expression of SIRT1 may be different from the results of *in vivo* experiments. The specific mechanism is not clear at present; this still needs more work. Finally, the precise mechanism by which irisin regulates the SIRT1/Nrf2 signal axis needs further investigation.

## Conclusion

In summary, as shown in Figure 7, our findings demonstrated that SA-AKI induced ferroptosis by increasing iron and lipid peroxidation. Irisin effectively suppressed ferroptosis and alleviated SA-AKI and improved the mitochondria function *via* induction of the SIRT1/Nrf2 signal axis. This study provides favorable evidence for the application of irisin in the treatment of SA-AKI.

## Data Availability

The original contributions presented in the study are included in the article/Supplementary Material; further inquiries can be directed to the corresponding authors.
