# Supplementary material for: Post-treatment With Irisin Attenuates Acute Kidney Injury in Sepsis Mice Through Anti-Ferroptosis via the SIRT1/Nrf2 Pathway
Source: Front Pharmacol. 2022 Mar 17;13:857067. doi: 10.3389/fphar.2022.857067 (PMC8970707; doi:10.3389/fphar.2022.857067)
Supplement: Supplementary file 1 [file DataSheet1.zip › supplement/zqysupplement.docx]

**Supplementary materials**

**Methods and materials**

Firstly, to construct LPS-induced injury model in HK-2 cells, we explored the appropriate concentration of LPS. HK-2 cells were treated with different concentrations of LPS (0 μg/mL, 1 μg/mL, 5 μg/mL, 10 μg/mL) for 22 h. The CCK-8 kit (Beyotime C0037) was used for the quantitation of viable cell number. The concentrations of malondialdehyde (MDA) and glutathione (GSH) in HK-2 cells were determined by the MDA assay kit (Beyotime, Wuhan, China), and GSH assay kit (Beyotime, Wuhan, China) following to the protocols of respective manufacturer. The results in figure S1A-C showed that the cell number and the concentration of GSH in cells was significantly decreased, and the concentration of MDA in cells was significantly increased when the concentration of LPS was higher than 10 μg/mL compared with the control group, so 10 μg/mL was chosen as the appropriate concentration of LPS.

Secondly, to investigate the appropriate concentration of irisin, HK-2 cells were treated with different concentrations of irisin(2nM, 4nM, 8nM) before 10 μg/mL LPS administration. The concentrations of malondialdehyde (MDA) and glutathione (GSH) in HK-2 cells were determined. The results in figure S2A-C indicated that the cell number and the concentration of GSH in cells was significantly increased, and the concentration of MDA in cells was significantly decreased when the concentration of irisin was higher than 8nM compared with the LPS group, so 8nM was chosen as the appropriate concentration of irisin.

**Supplementary figures and figure legends**
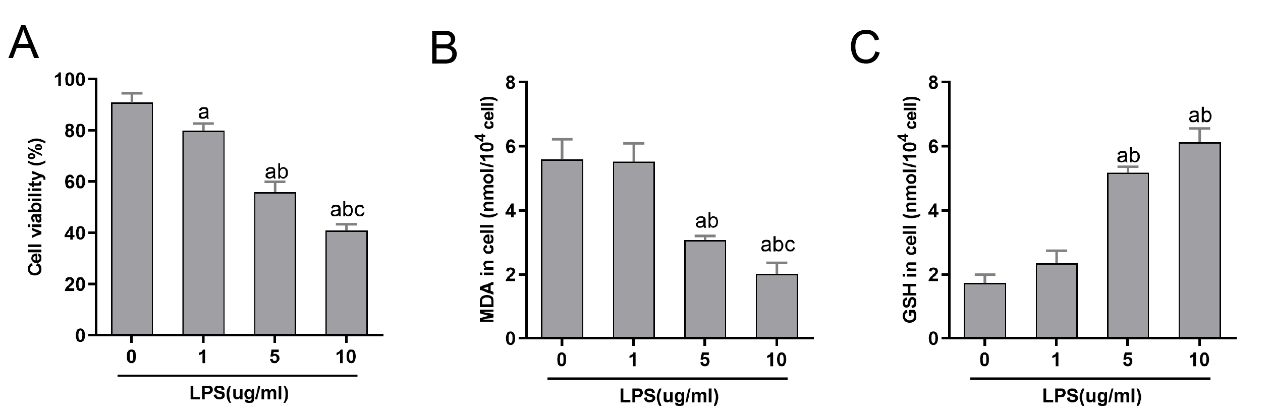


Figure S1. Preliminary experiments on the concentration of LPS treatment in vitro experiments. A: The number of live cells was detected using the CCK-8 kit after the treatment of HK-2 cells with different concentrations of LPS (0μg/mL, 1 μg/mL, 5μg/mL, 10 μg/mL) for 22 h. B: The levels of MDA in cells were analyzed using the MDA assay kit. C: The levels of GSH in cells were analyzed using the GSH assay kit. ^a^P < 0.05 vs. 0 group, ^b^P < 0.05 vs. 1 group, and ^c^P < 0.05 vs.5 group. The data are presented as mean ± SD (n = 3).


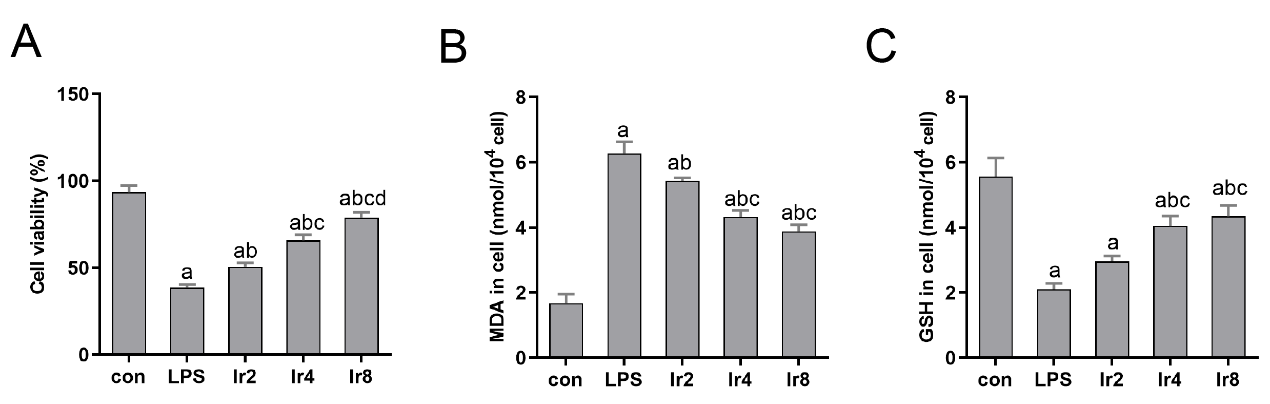


Figure S2. Irisin inhibited LPS induced cell injury dose-dependently. HK-2 cells were incubated with different concentrations of irisin (2nM, 4nM, 8nM) before 10 μg/mL LPS administration. A: Cell viability was determined using the CCK-8 assay kit. B: The levels of MDA in cells were analyzed using the MDA assay kit. C: The levels of GSH in cells were analyzed using the GSH assay kit. ^a^P < 0.05 vs. con group, ^b^P < 0.05 vs. LPS group, and ^c^P < 0.05 vs. Ir2 group, ^d^P < 0.05 vs. Ir4 group. The data are presented as mean ± SD (n = 3).
