# Supplementary figures and images for: Post-treatment With Irisin Attenuates Acute Kidney Injury in Sepsis Mice Through Anti-Ferroptosis via the SIRT1/Nrf2 Pathway
Source: Front Pharmacol. 2022 Mar 17;13:857067. doi: 10.3389/fphar.2022.857067 (PMC8970707; doi:10.3389/fphar.2022.857067)

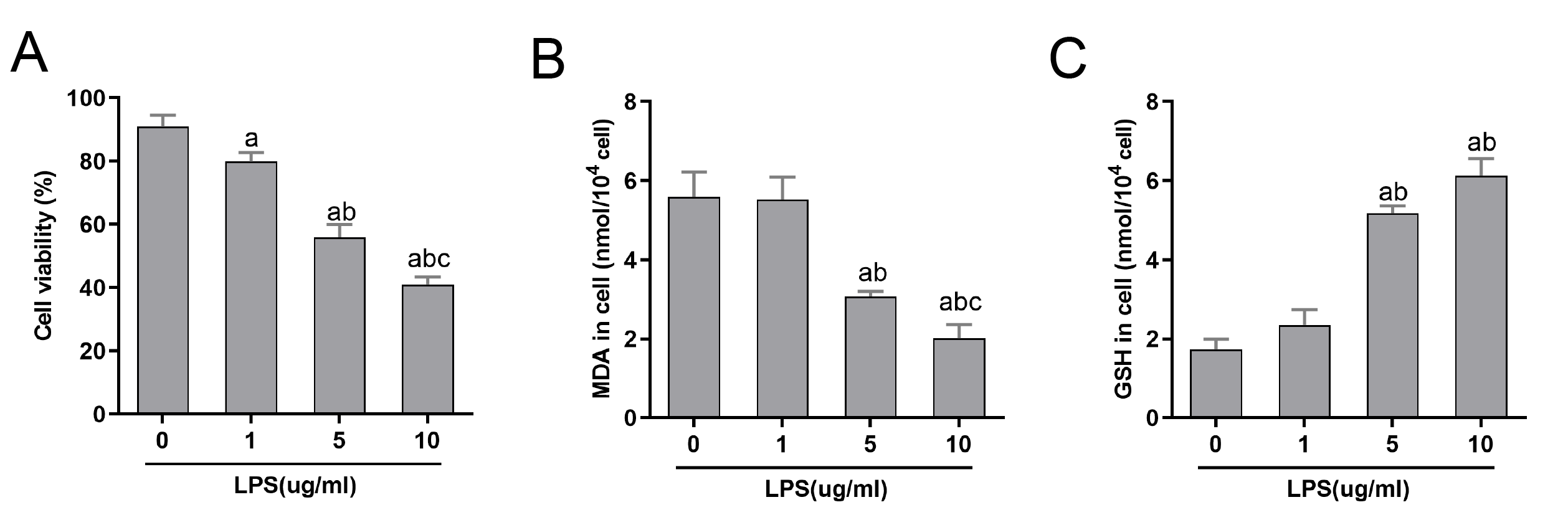

Supplement: Supplementary file 1 [file DataSheet1.zip › supplement/figS1/FigS1.tif]

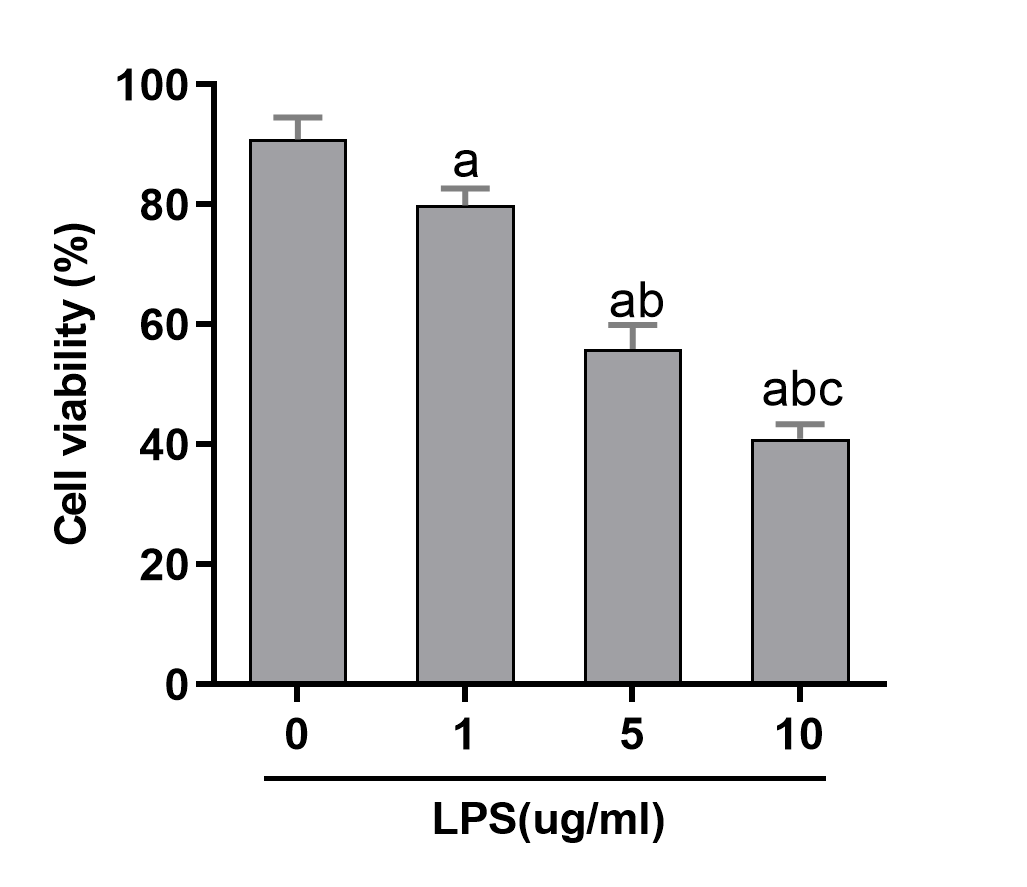

Supplement: Supplementary file 1 [file DataSheet1.zip › supplement/figS1/lps cell-1.tif]

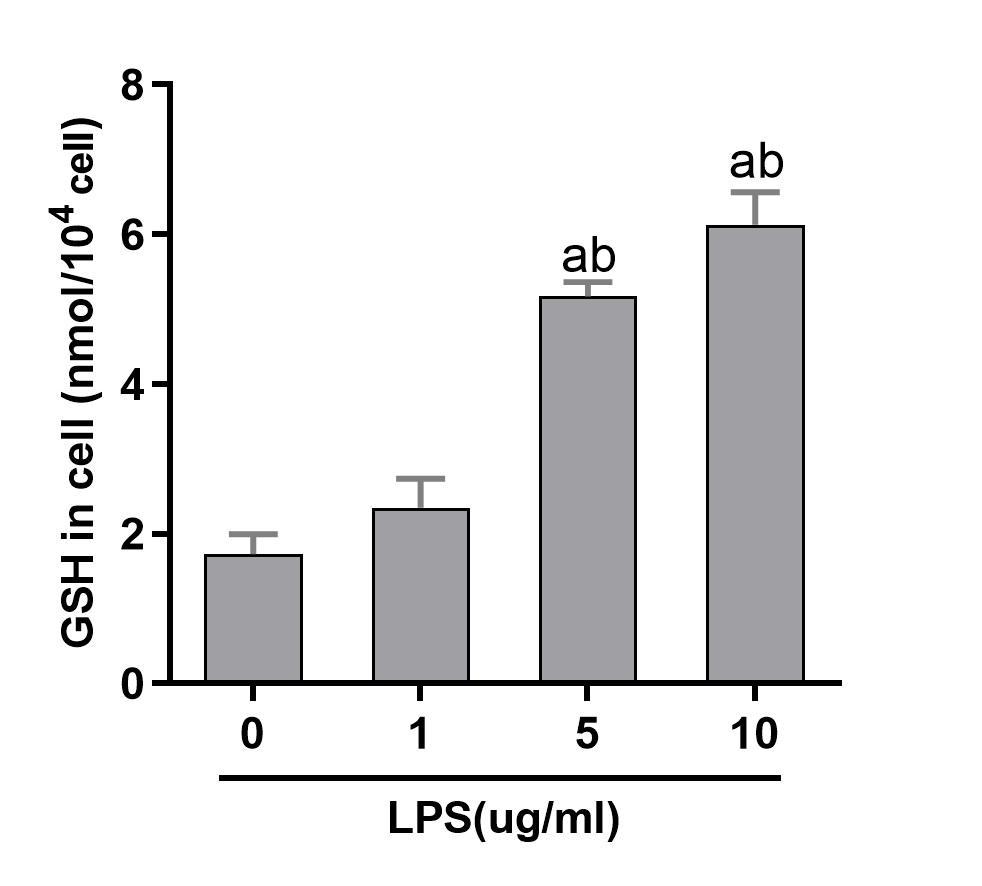

Supplement: Supplementary file 1 [file DataSheet1.zip › supplement/figS1/lpsgsh-1.tif]

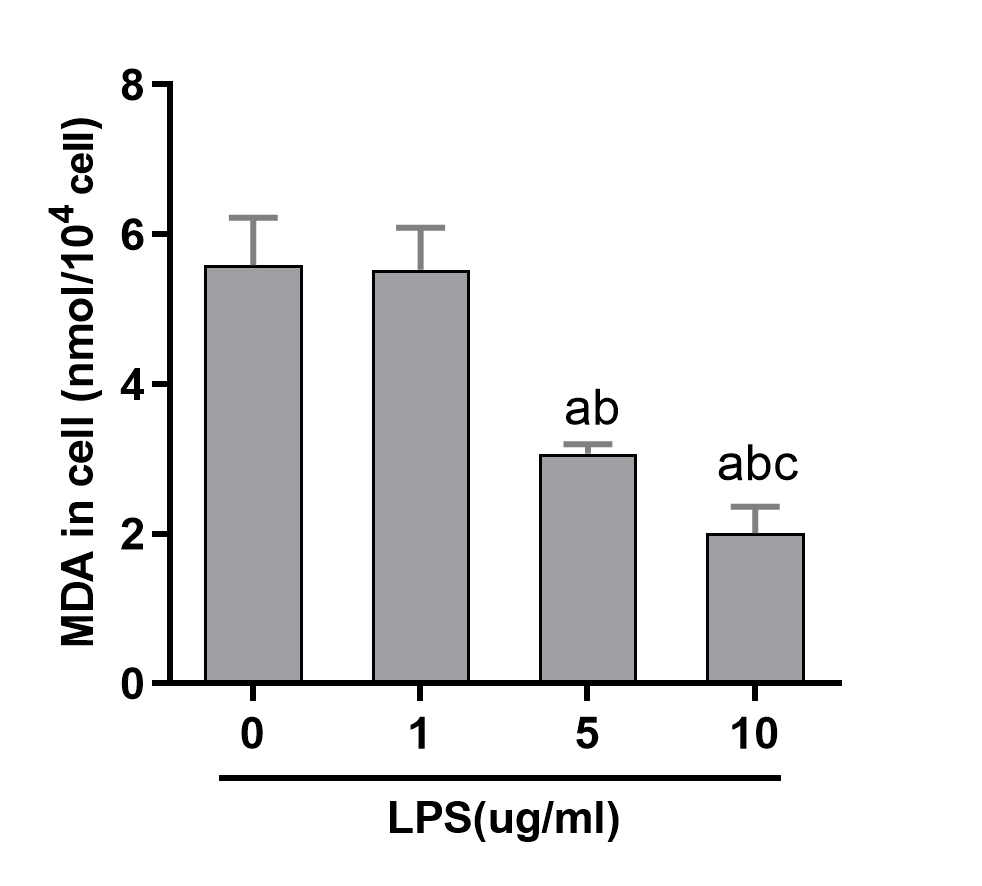

Supplement: Supplementary file 1 [file DataSheet1.zip › supplement/figS1/lpsmda-1.tif]

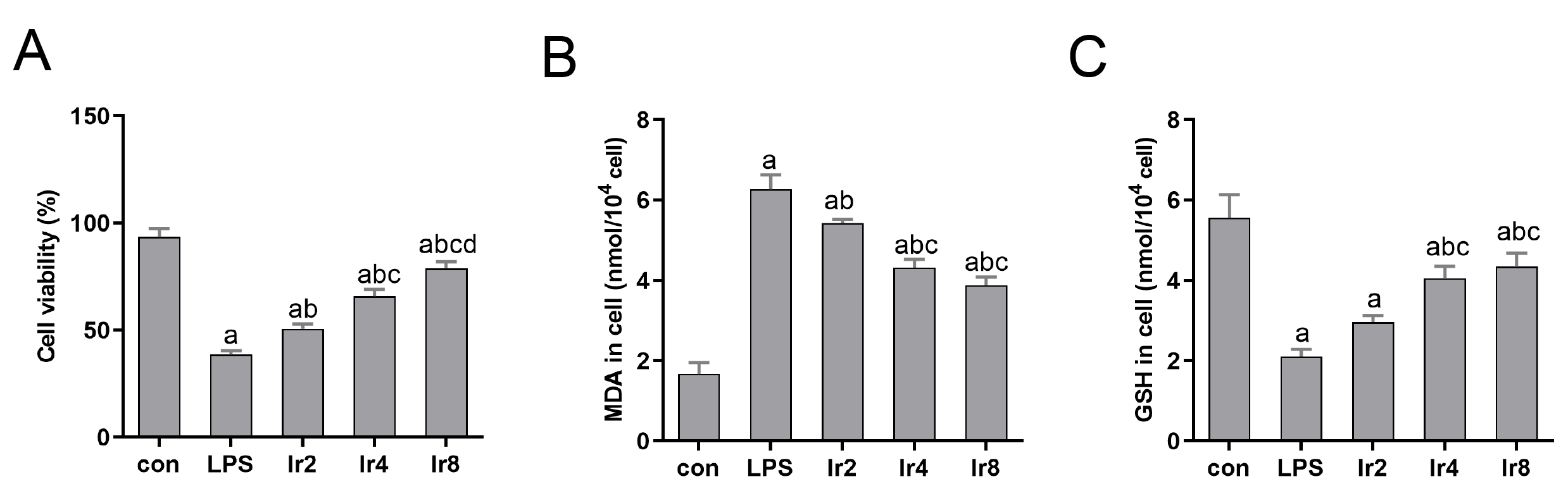

Supplement: Supplementary file 1 [file DataSheet1.zip › supplement/figS2/FigS2.tif]

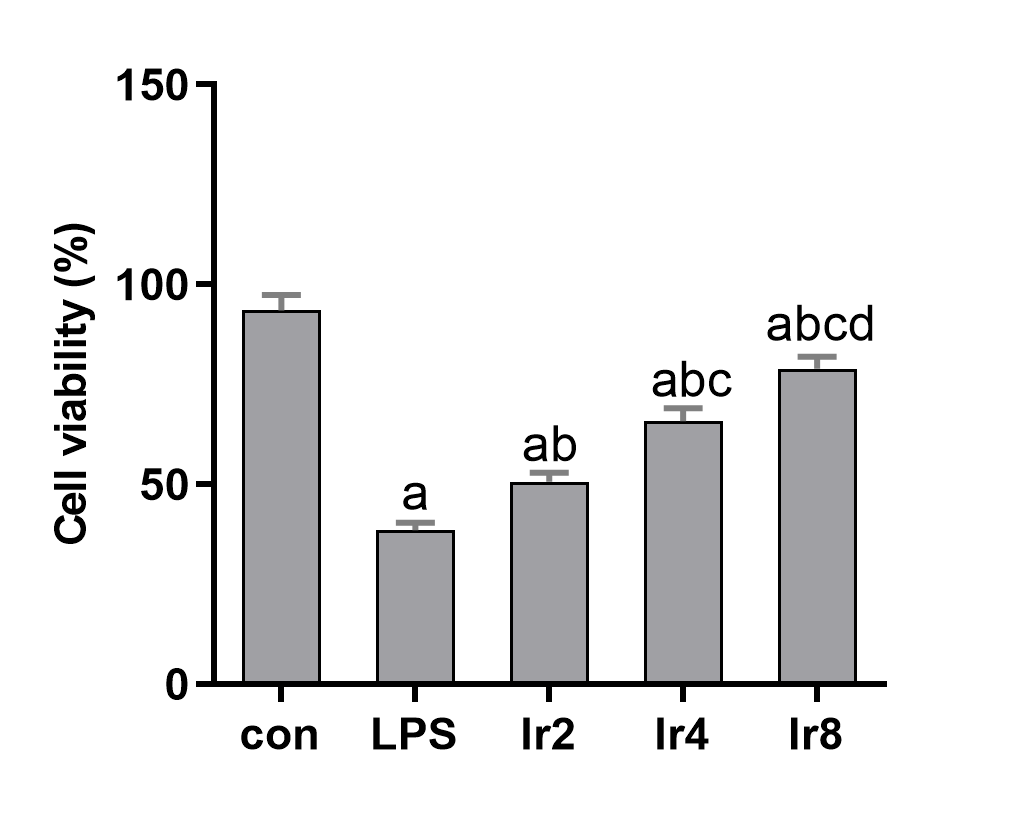

Supplement: Supplementary file 1 [file DataSheet1.zip › supplement/figS2/ir cellros.tif]

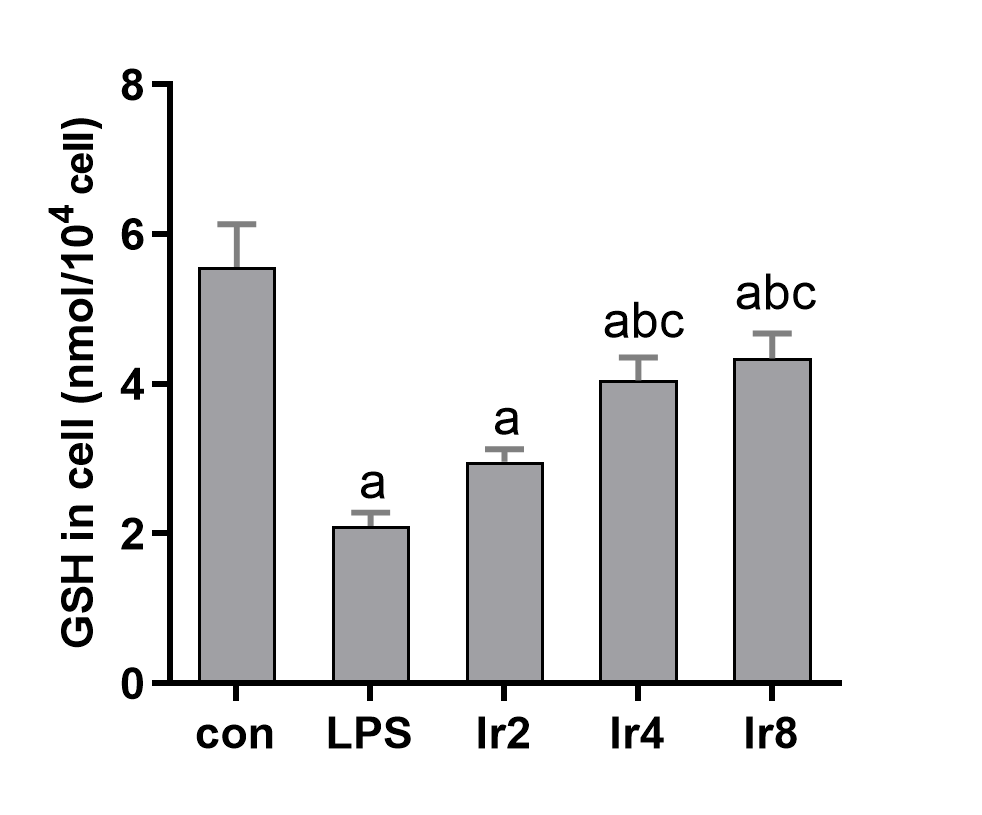

Supplement: Supplementary file 1 [file DataSheet1.zip › supplement/figS2/irgsh.tif]

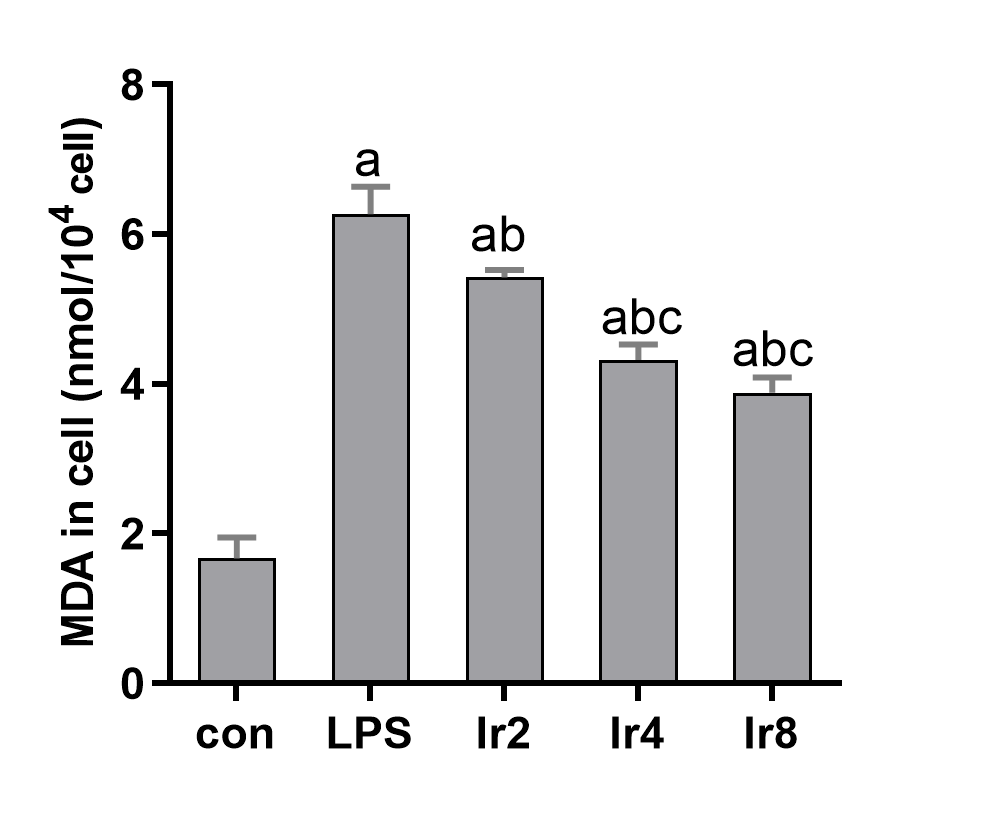

Supplement: Supplementary file 1 [file DataSheet1.zip › supplement/figS2/irmda.tif]
